# Preoperative planning and postoperative evaluation of total hip arthroplasty that takes combined anteversion

**DOI:** 10.1007/s00590-016-1777-8

**Published:** 2016-05-06

**Authors:** Hiroshi Imai, Joji Miyawaki, Tomomi Kamada, Jun Takeba, Naohiko Mashima, Hiromasa Miura

**Affiliations:** Department of Bone and Joint Surgery, Ehime University Graduate School of Medicine, Shitsukawa, Toon, Ehime 791-0295 Japan

**Keywords:** Developmental dysplasia of the hip, Total hip arthroplasty, The metaphyseal fit stem, Combined anteversion, Preoperative 3D-planning, The operative technique which prepares the socket first

## Abstract

The purpose of this study was to investigate whether postoperative combined anteversion (CA) can be kept within the safe zone while using cementless total hip arthroplasty (THA) using the operative technique which prepares the socket first for developmental dysplasia of the hip (DDH), by estimating the anteversion of the metaphyseal fit stem using preoperative three-dimensional (3D) computerized planning and by adjusting the anteversion of the socket using a navigation system that considers CA. Our subjects were 65 patients (65 hips) that had undergone cementless THA for DDH that could be observed for 1 year or more. Clinical assessments were made using the Japanese Orthopaedic Association’s (JOA) hip score. For a radiological evaluation, we investigated 3D-planned stem versions, postoperative stem versions, preoperative and postoperative CA, and the relationship between CA and dislocation tendencies with temporary intraoperative reductions. JOA hip scores improved from 52.3 ± 11.4 points to 88.9 ± 8.6 points. CT evaluations revealed that 3D-planned stem versions were strongly correlated with postoperative stem versions (*r* = 0.80; *p* < 0.01). Preoperative CA was 50.5° ± 7.2°, and postoperative CA was 41.3° ± 8.6°. Postoperative CA was kept within the safe zone in 61 hips. No intraoperative dislocation tendencies were observed in any hips. By estimating the anteversion of the cementless metaphyseal fit stem using 3D planning preoperatively and adjusting the angle of anteversion of the socket using a navigation system that considers CA intraoperatively, postoperative CA can very frequently be kept within the safe zone, even with cementless THA using the operative technique which prepares the socket first for DDH.

## Introduction

The optimum implant placement in cementless total hip arthroplasty (THA) is important to obtain stable, long-term clinical results. Conversely, implant malposition can cause postoperative prosthetic impingement, resulting in postoperative dislocation, implant failure, excessive polyethylene wear, and decreased postoperative range of motion [1]. Developmental dysplasia of the hip (DDH), which accounts for 80 % of hip osteoarthritis in Japanese patients, is associated not only with problems in the acetabulum but also with variations in femur morphology [2, 3]. In cases where cementless metaphyseal fit stems are fixed using the press-fit method, the stems are likely to induce strong anteversion or retroversion and cause prosthetic impingement [4]. Additionally, cementless THA using the operative technique which prepares the socket first can also cause prosthetic impingement. In order to prevent prosthetic impingement, the combined anteversion (CA) technique, which combines the anteversion of the femoral stem and the acetabular socket, has been revised in recent years [5, 6]. Furthermore, it is necessary to keep CA within the safe zone [7, 8]. In THA applying the CA technique, typically the stem is placed first, and the angle of the stem anteversion is fixed. Next, based on the angle of the stem anteversion, the angle of the socket anteversion is adjusted to avoid impingement during the surgery [9, 10]. However, the neck portion of many stem models often obstructs the acetabular socket placement using the operative technique which prepares the femoral stem first. To counteract this, we loaded preoperative computed tomography (CT) images into the Kyocera 3D-template^®^ (Kyocera Medical, Osaka, Japan), a THA planning software, and estimated the angle of the stem anteversion preoperatively and adjusted the angle of the socket anteversion so that CA was kept within the safe zone. Our target was to maintain the combined anteversion in the safe zone of 40° ± 15° [5–8, 10]. Intraoperatively, the socket was first placed at the planned angle of anteversion using the navigation system; then, the femoral stem was placed to fit into the bone marrow cavity of the femur [11–13]. The aim of this study was to investigate whether postoperative CA can be kept within the safe zone while using the operative technique which prepares the socket first in cementless THA for DDH, by estimating the anteversion of the metaphyseal fit stem using preoperative three-dimensional (3D) computerized planning and by adjusting the anteversion of the socket using a navigation system that considers CA.

## Materials and methods

**Fig. 1:**
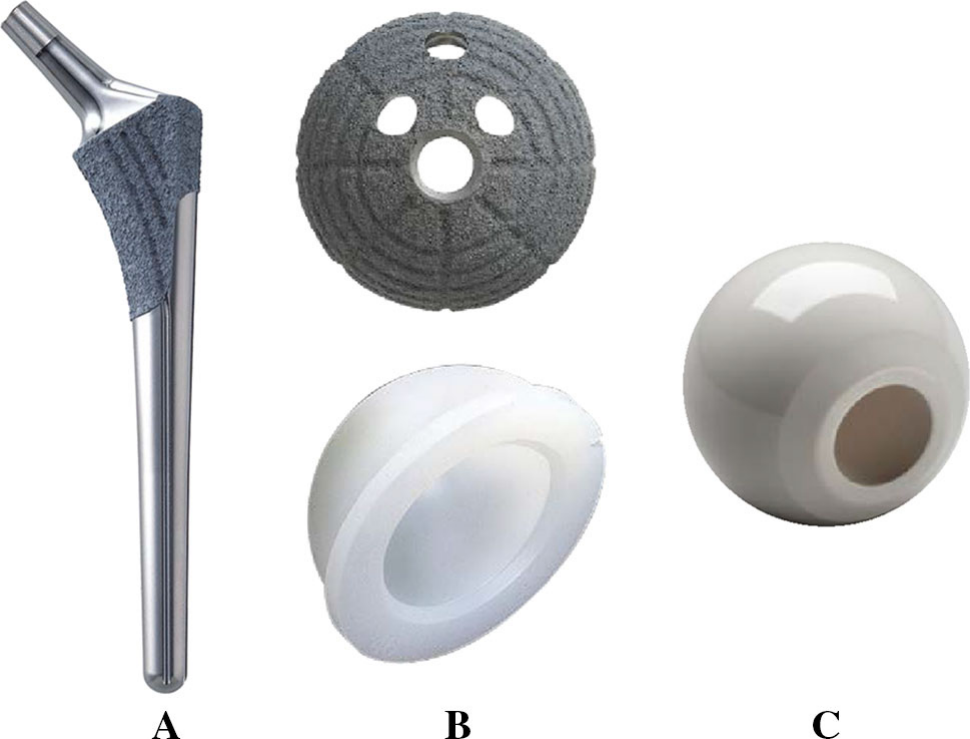
**a** HA coating metaphyseal fit-and-fill stem (Perfix 910^®^ HA stem Kyocera Medical). **b** HA coating socket and cross-linked polyethylene (AMS^®^ HA socket and Aeonian^®^ Kyocera Medical). **c** 28-/32-mm zirconium femoral head (Bioceram^®^ AZ209 Kyocera Medical)

**Fig. 2:**
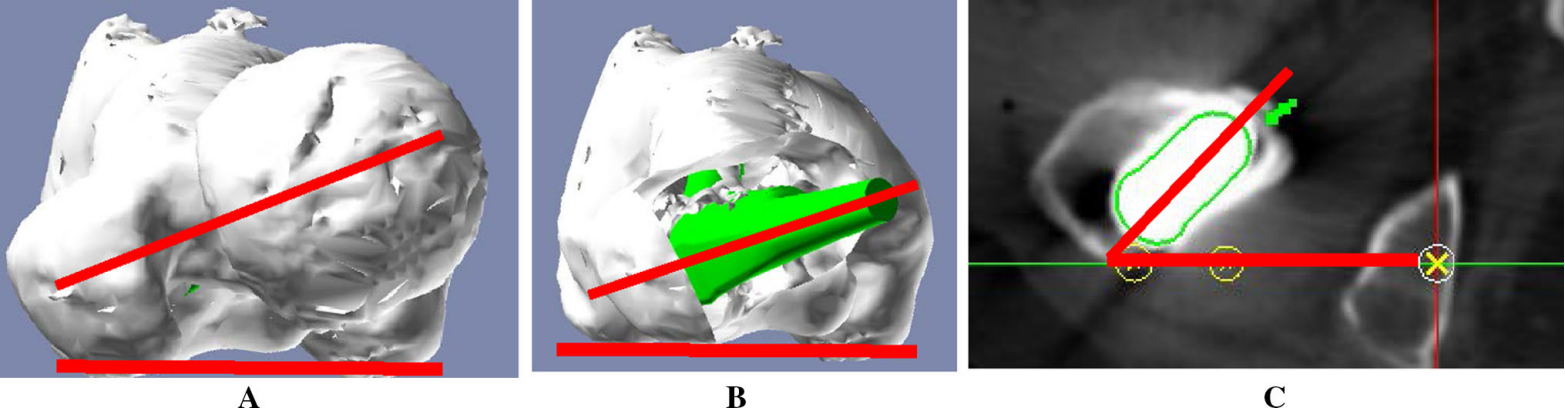
**a** Native femoral version was defined as the angle between the femoral neck axis and the table-top plane. **b** The 3D-planned stem version was defined as the angle between the 3D-planned stem axis and the table-top plane. **c** The postoperative stem version was defined as the angle between the postoperative stem axis and the table-top plane

**Fig. 3:**
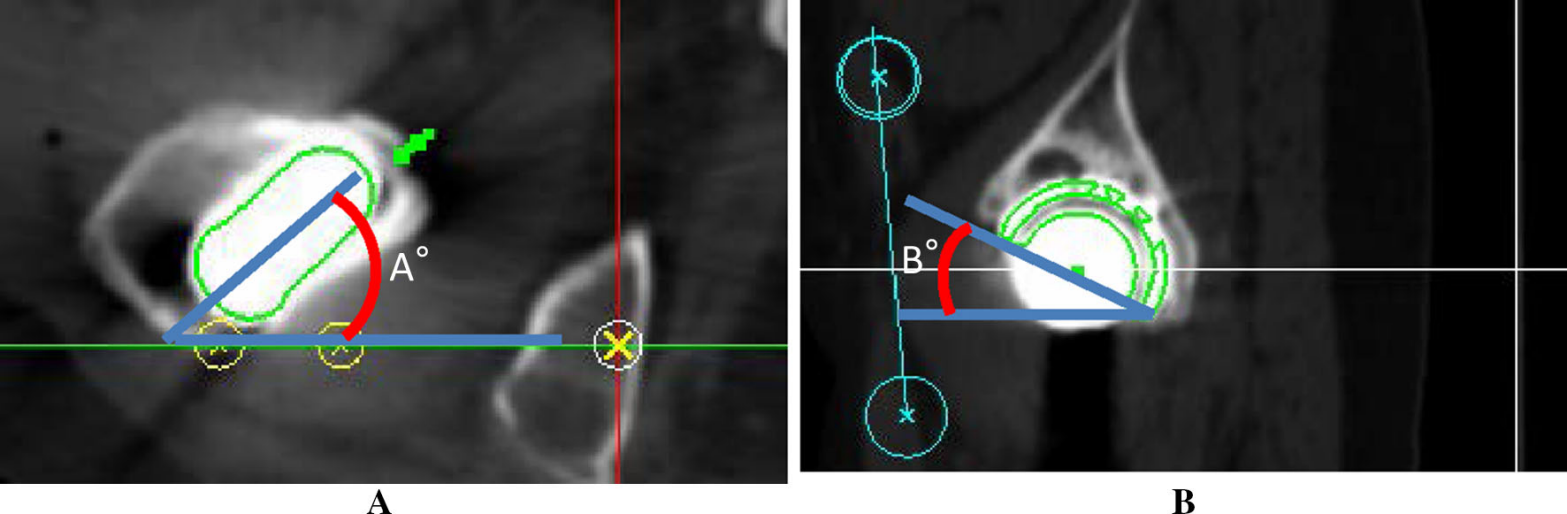
Combined anteversion = *A* + sin^−1^{sin (*B*) × cos (operative inclination)}. *A* = anteversion of the stem. *B* = operative anteversion of the socket. To obtain the socket anteversion angle, we measured the angle that the functional pelvic plane makes with the socket and converted the values into radiographic anteversion angle using the conversion equation described by Murray

Sixty-five patients (65 hips) were recruited for the study. They were selected out of 70 patients who underwent cementless THA using the operative technique which prepares the socket first and who could be observed for 1 year or more. The following cases were excluded: three hips that underwent THA for avascular necrosis of the femoral head and two hips that used the conical stem due to a greater than 55° anteversion angle of the stem as seen on preoperative 3D computerized planning. The mean age of the subjects at the time of surgery was 60.2 years [standard deviation (SD) 12.3 years, range 33–78 years]. There were three male patients (three hips) and 62 female patients (62 hips). The mean duration of follow-up after surgery was 1.3 years (SD 0.2, range 1.0–1.5 years). DDH was classified using the Hartofilakidis et al. [14] classification. Based on the Hartofilakidis et al. classification, 45 hips in our group had type A hip dysplasia, 20 had type B with low dislocation, and there were no cases of type C with high dislocation. We aimed for a greater than 5° angle of the socket center-edge (CE) [15]; therefore, twelve patients (12 hips) were managed with autologous morselized bone grafts in the gap between the host bone and the lateral margin of the socket in a dysplastic acetabulum. There was no case with autologous block bone grafts. Patient demographic data are summarized in Table 1. We used a hydroxyapatite (HA) coating metaphyseal fit-and-fill stem (PerFix 910^®^ HA stem; Kyocera Medical, Osaka, Japan), an HA coating socket (AMS^®^ HA socket; Kyocera Medical, Osaka, Japan), a 28/32-mm zirconium femoral head (Bioceram^®^ AZ209; Kyocera Medical, Osaka Japan), and a cross-linked polyethylene liner (Aeonian^®^; Kyocera Medical, Osaka, Japan) as shown in Fig. 1. We used the Kyocera 3D-template^®^ to keep CA in the safe zone of 40° ± 15°. The cases that had a planned stem version of more than 50° in the 3D planning software-based CT were targeted to have CA less than 55°, while the cases that had a planned stem anteversion angle of less than 0° were targeted to have CA of more than 25°. The rest were targeted within the angle range of 30°–50°. As a result, our target was to maintain the combined anteversion in the safe zone of 40° ± 15° [5–8, 10]. The details of the operations are as follows. The surgery was performed by one senior surgeon. All operations were performed while the patient was in the lateral position using a modified Hardinge approach [16]. After the initial skin incision, preparation of the socket was conducted. For socket placement, we used a CT-based fluoroscopy matching navigation system (VectorVision Hip 3.5.1; BrainLAB, Feldkirchen, Germany). A ball used for the infrared light navigation system was fixed with two pins onto the iliac crests. The acetabulum was under-reamed by 1 mm. Next, a socket of the planned size was fixed using the press-fit method. The insertion angles were set to 40° for radiographic inclinations and to the planned angles for radiographic anteversions, which were adjusted based on the angle of anteversion of the stem based on the 3D-template^®^ analysis. To achieve the initial fixation, a few screws were used and morselized autologous bone grafts were placed against the superolateral part of the ilium above the acetabular socket in twelve patients (12 hips). Subsequently, according to the anatomical configuration of the femur, the stem was fixed using the press-fit method in order to achieve a strong initial fixation with the bone. After placing the implant, we verified that there was no tendency to dislocate with a hip in flexion of 90°, adduction of 20°, internal rotation of 40°, extension of 20°, and external rotation of 40°. When any dislocation tendencies were observed, a polyethylene liner with a lip was utilized. After verifying that the dislocation tendency was improved, a drainage tube was placed and the wound was closed. On postoperative day 1, if patients were in good overall clinical condition, the drain was removed and gait training with full weight bearing was initiated. Clinical assessment was completed twice by two of the orthopedic surgeons, each of whom had more than 15 years of experience in assessing hip function. The time between measurements was at least 2 weeks. Both were blinded to the radiographic results at the time of the evaluation. They used the Japanese Orthopaedic Association’s standard for evaluation of hip joint function (JOA’s hip score) [17] and investigated the incidence of postoperative complications. The JOA’s hip score is a 100-point scale that comprises of the following subcategories: pain (0–40 points), ability to walk (0–20 points), range of motion (0–20 points), and ability to complete daily living tasks (0–20 points). The hip flexion angle was measured with the patient in a supine position with the contralateral lower extremity fixed to the table with 0° of rotation in both lower extremities to prevent compensation with pelvic extension. Then, the hip abduction angle was measured with the patient in a supine position with the contralateral lower extremity fixed in maximum abduction to prevent compensation by pelvic tilting. Higher scores indicated better conditions. Scores at the final follow-up were compared to preoperative scores. We assessed the fixation of the socket and the stem. Radiolucent lines and osteolytic lesions in the three acetabular zones of DeLee and Charnley were recorded [18]. Socket migration was defined as a change in the position of the acetabular component of more than 2 mm or a change in socket inclination of more than 5° [19]. The femoral stem was evaluated with regard to the presence of radiolucent lines, osteolysis, cancellous condensation, cortical hypertrophy, reactive lines, and pedestal formations according to the criteria set by Engh et al. [20]. In addition, as part of the CT evaluation, we investigated the native femoral angle of anteversion (native femoral version), the angle of anteversion of the stem on preoperative 3D computerized planning (3D-planned stem version), the postoperative angle of anteversion of the inserted stem (postoperative stem version; Fig. 2), the insertion angle of the socket (Fig. 3), the preoperative planned and postoperative CA, the relationship between an intraoperative dislocation tendency and postoperative CA, and the relationship between the postoperative anteversion of the socket, the postoperative stem version, and keeping within the safe zone of the postoperative CA. For the hip joint coordinate system [21], the plane that connects both anterior superior iliac spines and the pubic symphysis was defined as the anterior pelvic plane. The table-top plane was defined as the functional pelvic plane [22]. To obtain the socket inclination and anteversion angles, we measured the angle that the functional pelvic plane makes with the socket and converted the values into radiographic inclination and anteversion angles using the conversion equation described by Murray [23]. In addition, the coordinate system for the femur was defined as follows: the plane that connects the most posterior points of the medial and lateral condyles, and the most posterior point of the greater trochanter (table-top plane) was defined as the reference plane for the femur. The line connecting the piriform fossa of the femur and the center of the knee was defined as the femoral axis. The axis constructed by projecting the femoral axis to the femoral reference plane was defined as the Z-axis. We measured the native femoral version, the 3D-planned stem version, and the postoperative stem version. All radiographic measurements were performed by the same observer. 3D CT scans were performed using a Philips Brilliance 64 scanner (Marconi Medical System, Best, Netherlands). The scanning technique parameters were: 120 kV, 150–250 effective mAs (depending on the patient’s size), and 0.5 s rotation time. Contiguous slices (2.0 mm) were obtained from the bilateral anterior superior iliac spines to the distal end of the femur, with the patient in a supine position with the hips extended and thighs horizontal and parallel. All raw CT scan data in Digital Imaging and Communications in Medicine (DICOM) format were entered into an available planning software for the Kyocera 3D-template^®^.

**Table 1:** Patient demographics

Gender	
Male	3
Female	62
Age, mean (range)	60.2 ± 12.3 (33–78)
BMI, mean (range)	24.6 ± 3.9 (17.4–35.6)
Duration, mean (range)	1.3 ± 0.2 (1.0–1.5)
Hartofilakidis classification	
Type A	45
Type B	20
Type C	0
Morselized bone grafts	
No	53
Yes	12

### Statistical analysis

The normality of the continuous data was assessed with Levene’s test. Since the data were distributed normally, the unpaired Student’s *t* test was used. Intraobserver variances for the JOA’s hip score were determined by comparing separate assessments of the same patient by the same observer with at least a 2-week interval between assessments. Intraobserver and interobserver variances in the JOA’s hip score were expressed using interclass correlation coefficients (ICC) with ICC < 0.20 indicating slight agreement, 0.21–0.40 fair agreement, 0.41–0.60 moderate agreement, 0.61–0.80 substantial agreement, and >0.80 almost perfect agreement [24]. The relationship between the native femoral version and the postoperative stem version, the 3D-planned stem version and the postoperative stem version was examined using correlation analysis. The relationship between the radiographic anteversion of the socket, the postoperative stem version, and adherence to the safe zone of postoperative CA was investigated using a scatter diagram. SPSS for Windows version 20 (IBM, Armonk, NY, USA) was used for all statistical analyses. A *p* value <0.05 was used to indicate statistical significance.

## Results

**Fig. 4:**
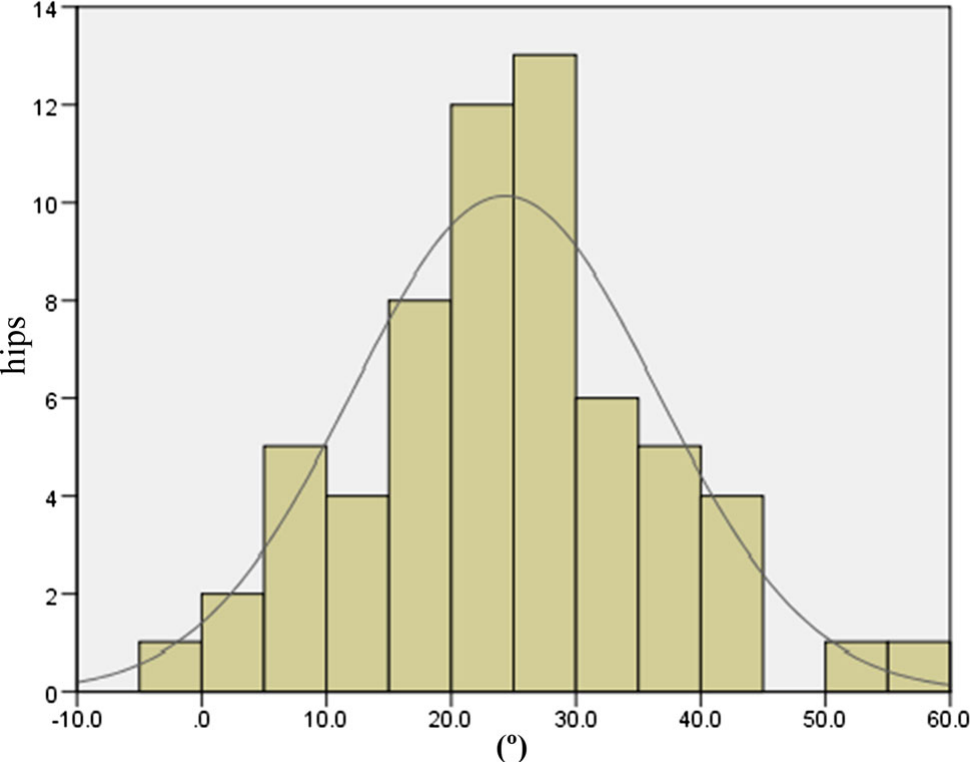
Distribution of the native femoral version

**Fig. 5:**
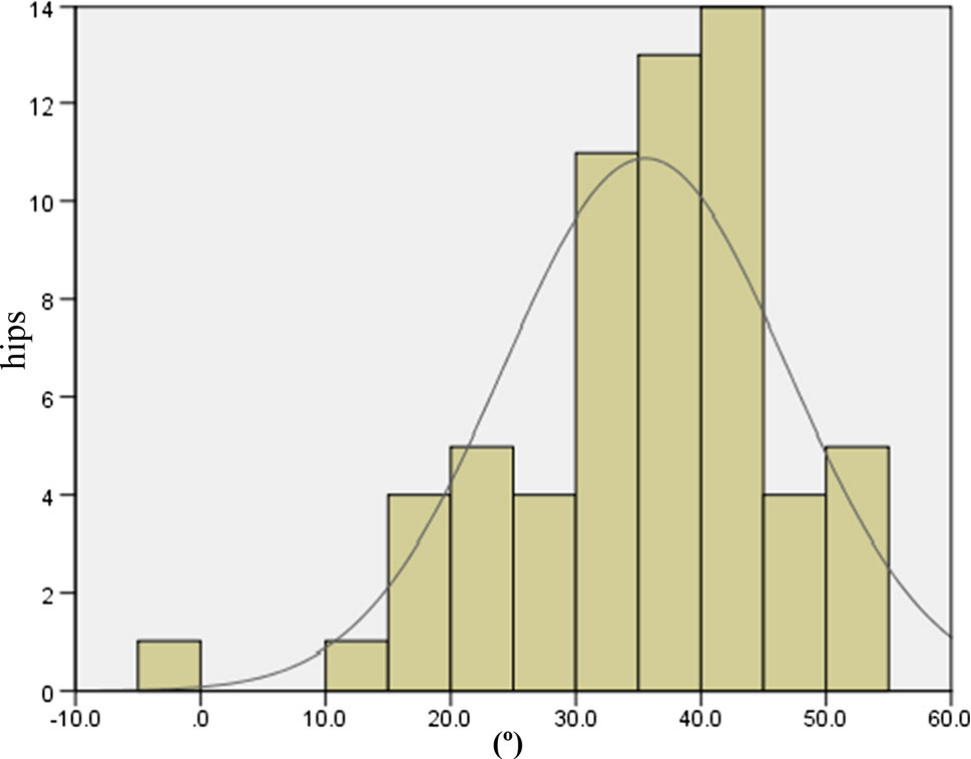
Distribution of the 3D-planned stem version

**Fig. 6:**
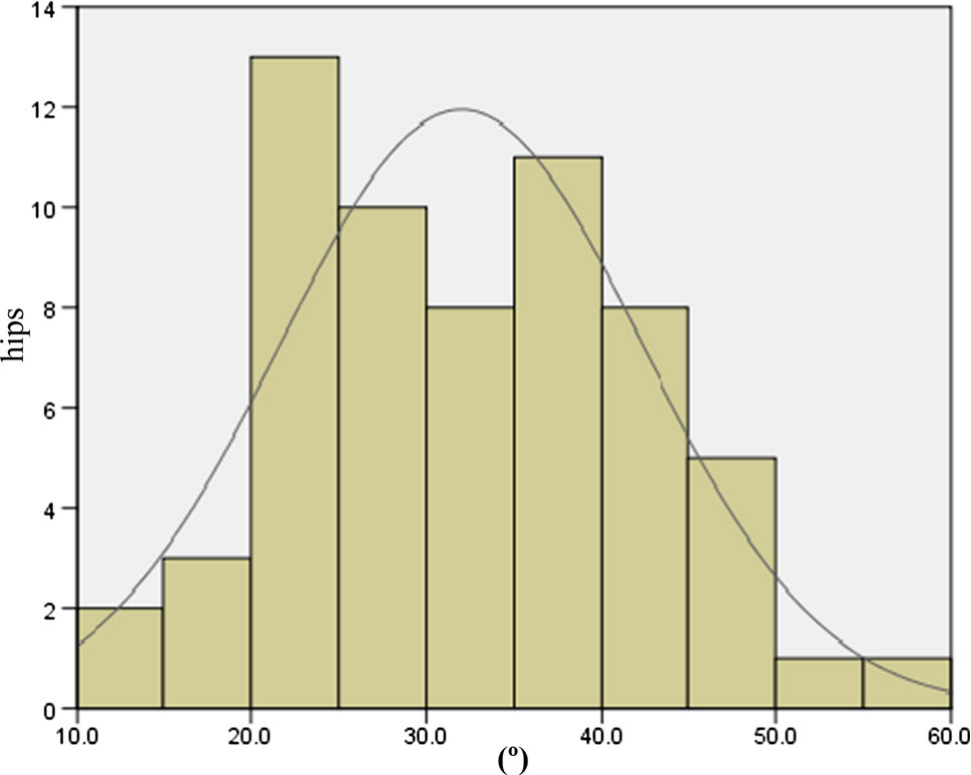
Distribution of the postoperative stem version

**Fig. 7:**
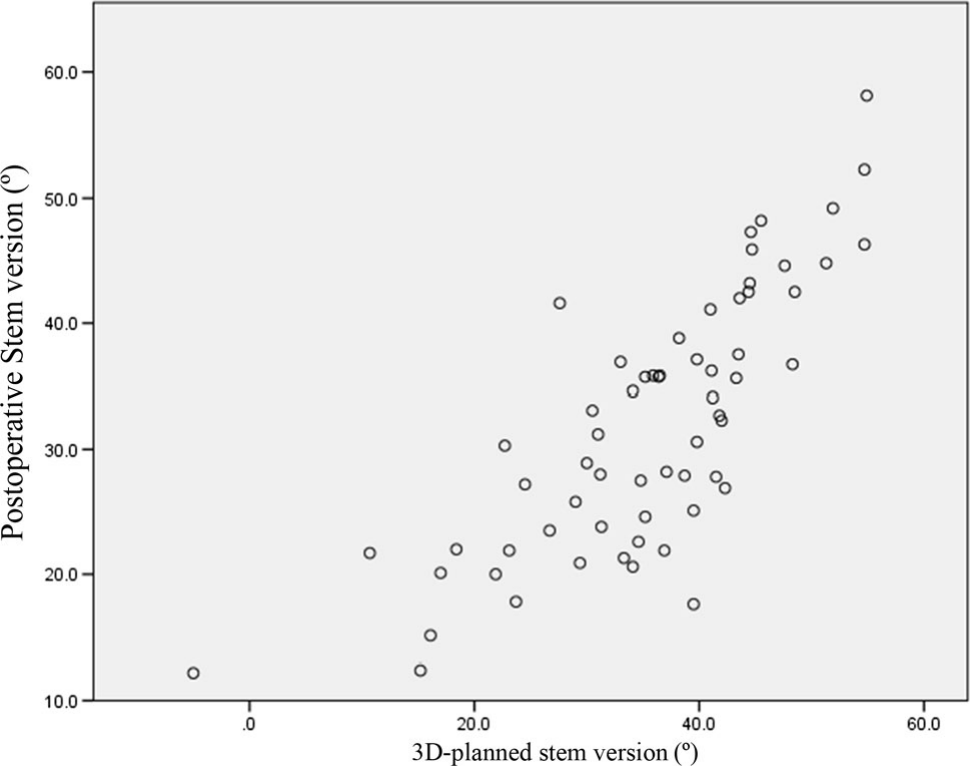
Relationship between the 3D-planned and the postoperative stem version

**Fig. 8:**
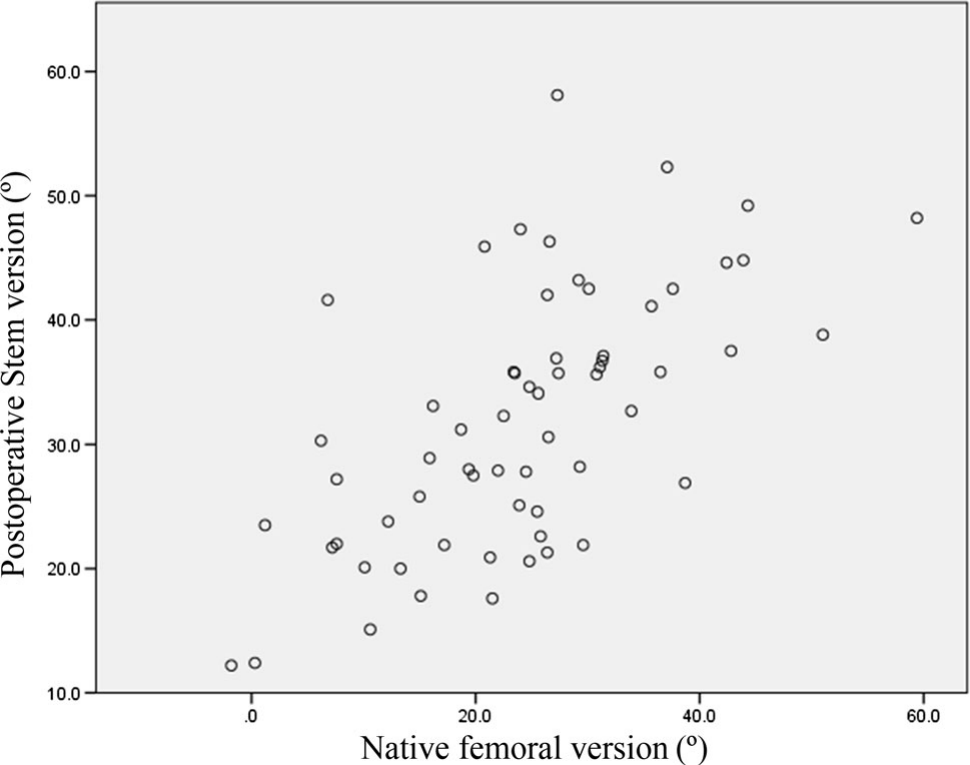
Relationship between the native femoral and the postoperative stem version

**Fig. 9:**
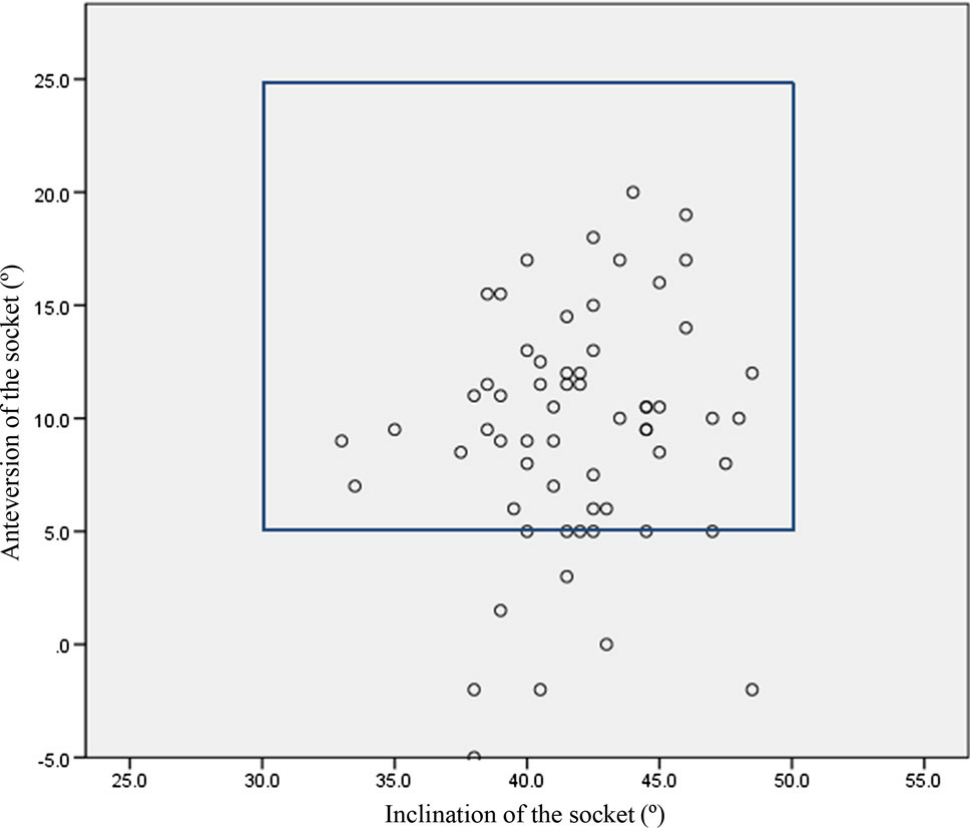
Radiographic angle of the socket. In 58 of 65 hips, radiographic inclination and anteversion of the sockets were kept within the safe zone proposed by Lewinnek et al.

**Fig. 10:**
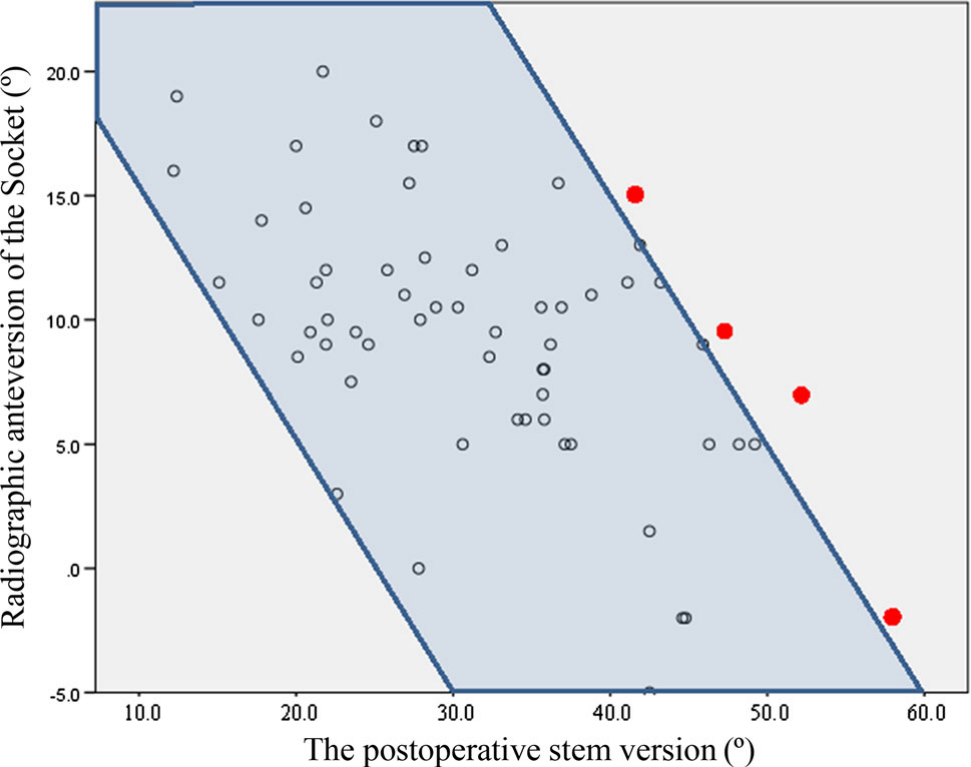
Scatter diagram of the radiographic anteversion of the socket and the postoperative stem version 61 hips within the shaded area represent the postoperative CA within the safe zone. The four hips shown in red had postoperative CA out of the safe zone

The JOA hip scores improved from 52.3 points (SD 11.4, range 24–79) preoperatively to 88.9 points (SD 8.6, range 63–99) postoperatively. Two intraobserver ICCs were calculated; both were 0.98. The interobserver variance had an ICC of 0.86. These values indicate almost perfect agreement with the JOA’s hip score as measured during physical examinations. None of the patients developed postoperative infections, paralysis, deep vein thrombosis, or dislocation. In the radiological evaluations, no radiolucent lines were observed. No periacetabular osteolysis in any of the three DeLee and Charnley zones was detected in any of the sockets for the entire follow-up period. No socket migration was observed in any hips. No radiolucent lines and osteolysis at the bone–stem interfaces and no subsidence or loosening were evident in any of the radiographs. All autologous morselized bone grafts in the sockets incorporated without collapse and without resorption. In the Kyocera 3D-template^®^ analysis, the native femoral version was 24.3° (SD 12.2°, range −1.8° to 59.4°; Fig. 4), the 3D-planned stem version was 35.6° (SD 11.4°, range −5.0° to 54.9°; Fig. 5), and the postoperative stem version was 32.0° (SD 10.3°, range 12.2°–58.1°; Fig. 6). The 3D-planned stem version was strongly correlated with the postoperative stem version (*r* = 0.80; *p* < 0.01; Fig. 7). The difference in the mean 3D-planned stem version and the postoperative stem version was −3.4° (SD 7.1°, range −21.9° to 17.2°). On the other hand, the native femoral version was only relatively weakly correlated with the postoperative stem version (*r* = 0.63; *p* < 0.01; Fig. 8), with a 7.7° (SD 9.6°, range −12.2° to 34.8°) difference. The postoperative insertion angles of the sockets were as follows: radiographic inclination, 41.9° (SD 3.4°, range 33°–48.5°), and radiographic anteversion, 10.7° (SD 6.5°, range −2.7° to 31.8°). In 58 of 65 hips, radiographic inclination and anteversion of the sockets were kept within the safe zone proposed by Lewinnek et al. [25] (Fig. 9). The rest had radiographic inclinations of 38.0, 38.0, 39.0, 40.5, 41.5, 43.0, and 48.5 and radiographic anteversions of −5.0, −2.0, 1.5, −2.0, 3.0, 0, and 2.0, respectively. The preoperative 3D-planned CA was 50.5° (SD 7.2°, range 25.0°–55.0°), and the CA measured on the postoperative CT images loaded into the Kyocera 3D-template^®^ was 41.3° (SD 8.6°, range 25.6°–59.3°), which was 9.1° (SD 10.1°, range −24.1° to 29.9°) less than the preoperative planned CA. There were 61 hips (93.8 %) in which the postoperative CA was within the safe zone and four hips in which the CA fell out of the safe zone on postoperative images. Specifically, four hips had a CA of over 55° (56.1°, 56.6°, 56.8° and 59.3°). In three hips, the CA was due to a stem anteversion (41.6°, 47.3°, 58.1°) that was unexpectedly larger than the 3D-planned stem version (27.6°, 44.6°, 54.9°, respectively) had predicted. The fourth hip had an unexpectedly larger socket anteversion (7.0°), which needed to be adjusted to a socket anteversion of less than 0.3° to achieve the 3D-planned stem version of 54.7°. There was one hip which showed 25.0° of CA on preoperative images: This case had retroversion of the femur (native femoral version = −1.8°, 3D-planned stem version = −5.0°). Therefore, it could be kept within the safe zone of the postoperative CA, resulting in a socket anteversion angle of 30° intraoperatively. We also evaluated the dislocation tendencies with temporary intraoperative reductions in 65 hips based on the operative reports. No intraoperative dislocation tendencies were observed in any hips. Therefore, the polyethylene liner with a lip was not utilized. The relationship between the radiographic anteversion of the socket, the postoperative stem version, and adherence to the safe zone of postoperative CA is indicated using a scatter diagram (Fig. 10). A total of 61 hips within the shaded area represent the postoperative CA within the safe zone. The four hips shown in red had postoperative CA out of the safe zone.

## Discussion

DDH, which accounts for 80 % of hip osteoarthritis among Japanese patients, is associated not only with problems in the acetabulum but also with variations in femur morphology [2, 3]. In cases where cementless metaphyseal fit stems are fixed using the press-fit method, the stems are likely to induce anteversion or retroversion and cause prosthetic impingement [4]. Additionally, cementless THA using the operative technique which prepares the socket first can also cause prosthetic impingement. In order to prevent prosthetic impingement, the CA technique, which combines anteversion of the femoral stem and the acetabular socket, has been proposed in recent years [5, 6]. The CA technique is especially useful for cementless THA using the operative technique which prepares the stem first, but there are concerns that the stem may be an obstacle in the operating field on the acetabular side while using this approach. Similarly, the infrared light navigation system for placing the socket positioned on the ilium may obstruct the stem insertion process while performing THA using the direct lateral approach [16]. To counteract this, we preoperatively calculated the 3D-planned stem version using the Kyocera 3D-template^®^ based on CT images for THA and used the CA technique in cementless THA using the operative technique which prepares the socket first. Previously, Mckibbin et al. [26] reported that the sum of acetabular and femoral anteversion ranged from 30° to 40° in their cadaver study. Subsequently, the values for the safe zone in CA were reported by Widmer et al. [7] and Yoshimine et al. [8] in their mathematical studies. We often observe that strong anteversion or retroversion occurs in DDH patients with THA that uses cavity-occupying cementless stems. The cases with planned stem version angles of more than 50° in the 3D planning software-based CT were targeted to have less than 55° of the CA, while the cases with planned stem anteversion angles of less than 0° were targeted to have more than 25° of the CA. The rest were targeted to be within the angle range of 30°–50°. As a result, our goal was to maintain the combined anteversion in the safe zone of 40° ± 15° [5–8, 10]. This study shows that by generating preoperative 3D images that consider CA, 3D-planned stem versions can provide a highly accurate estimate of postoperative stem versions. Moreover, in addition to positioning the socket in the estimated angle of anteversion, using a navigation system allows for control of CA within the safe zone over 90 % of the time. On the other hand, there was a difference of 7.7° (SD 9.6°, range −12.2° to 34.8°) between native femoral versions and postoperative stem versions, showing that merely considering the native femoral version is not sufficient for estimating the postoperative stem version [27, 28]. Dorr and Berry reported that dislocation after THA was induced not only by prosthetic impingement but also by other factors, for example soft tissue imbalance, the surgical approach, the patient’s education, and appearance of bone-to-bone impingement before prosthetic impingement [29, 30]. We were able to prevent the intraoperative dislocation tendency and postoperative dislocation by using preoperative 3D planning, considering CA. It is important to consider CA in order to avoid prosthetic impingement and dislocation after THA for DDH that has morphological differences on the femoral side, by estimating the stem version using 3D computerized planning and by adjusting the angle of anteversion of the socket using a navigation system that considers CA. By doing this, it is possible to prevent prosthetic impingement even in cementless THAs for DDH using the operative technique which prepares the socket first. The problems in this study include the lack of consideration of the following factors: (1) Two hips that used a conical stem due to a greater than 55° angle of 3D-planned femoral anteversion with the Kyocera 3D-template^®^ were excluded, (2) our conclusions are limited due to the small number of cases (*n* = 65), and (3) the retrospective design of the study, without a control group, only yields an evidence grade of IV for this report.

## Conclusions

The postoperative stem version of the metaphyseal fit stem can be estimated based on preoperative 3D computerized planning. Regarding morphological differences on the femoral side associated with DDH, by estimating these differences using preoperative 3D computerized planning and by adjusting the angle of anteversion of the socket using a navigation system that considers CA, postoperative CA can very frequently be kept within the safe zone, even with cementless THA using the operative technique which prepares the socket first.

## References

[1] Malik A, Maheshwari A, Dorr LD (2007). Impingement with total hip replacement. J Bone Joint Surg Am.

[2] Sugano N, Noble PC, Kamaric E, Salama JK, Ochi T, Tullos HS (1998). The morphology of the femur in developmental dysplasia of the hip. J Bone Joint Surg Br.

[3] Noble PC, Kamaric E, Sugano N, Matsubara M, Harada Y, Ohzono K, Paravic V (2003). Three-dimensional shape of the dysplastic femur: impingements for THR. Clin Orthop Relat Res.

[4] Jingwei Z, Wang L, Mao Y, Huiwu L, Ding H, Zhu Z (2014). The use of combined anteversion in total hip arthroplasty for patients with developmental dysplasia of the hip. J Arthroplasty.

[5] Dorr LD, Malik A, Dastane M, Zhinian Wan (2009). Combined anteversion technique for total hip arthroplasty. Clin Orthop Relat Res.

[6] D’Lima DD, Urquhart AG, Buehler KO, Walker RH, Colwell CW (2000). The effect of the orientation of the acetabular and femoral components on the range of motion of the hip at different head-neck ratio. J Bone Joint Surg Am.

[7] Widmer KH, Zurfluh B (2004). Compliant positioning of total hip components for optimal range of motion. J Orthop Res.

[8] Yoshimine F (2006). The safe-zones for combined cup and neck anteversions that fulfill the essential range of motion and their optimum combination in total hip replacements. J Biomech.

[9] Renkawitz T, Haimerl M, Dohmen L, Gneiting S, Wegner M, Ehret N, Buchele C, Schubert M, Lechler P, Woermer M, Sendtner E, Schuster T, Ulm K, Springorum R, Grifka J (2011). Minimally invasive computer-navigated total hip arthroplasty, following the concept of femur first and combined anteversion: design of a blinded randomized controlled trial. BMC Musculoskelet Disord.

[10] Nakashima Y, Hirata M, Akiyama M, Itokawa T, Yamamoto T, Motomura G, Ohishi M, Hamai S, Iwamoto Y (2014). Combined anteversion technique reduced the dislocation in cementless total hip arthroplasty. Int Orthop.

[11] DiGioia AM, Plakseychuk AY, Levison TJ, Jaramaz B (2003). Mini-incision technique for total hip arthroplasty with navigation. J Arthroplasty.

[12] Wan Z, Malik A, Jaramaz B, Chao BSL, Dorr LD (2009). Imaging and navigation measurement of acetabular component position in THA. Clin Orthop Relat Res.

[13] Ryan JA, Jamili AA, Bargar WL (2010). Accuracy of computer navigation for acetabular component placement in THA. Clin Orthop Relat Res.

[14] Hartofilakidis G, Stamos K, Karachalios T, Ioannidis TT, Zacharakis N, Greece A (1996). Congenital hip disease in adults. classification of acetabular deficiencies and operative treatment with acetabuloplasty combined with total hip arthroplasty. J Bone Joint Surg Am.

[15] Sugano N, Nishii T, Nakata K, Masuhara K, Takaoka K (1995). Polyethylene socket and almina ceramic heads in cemented total hip arthroplasty. A ten-year study. J Bone Joint Surg Br.

[16] Hardinge K (1982). The direct lateral approach to the hip. J Bone Joint Surg Br.

[17] Shima Y (1971). Standard for evaluation of osteoarthritis of the hip. J Jpn Orthop Assoc.

[18] DeLee JG, Charnley J (1976). Radiological demarcation of cemented sockets in total hip replacement. Clin Orthop Relat Res.

[19] Callaghan JJ, Dysart SH, Savory CG (1988). The uncemented porous-coated anatomic total hip prosthesis. Two-year results of a prospective consecutive series. J Bone Joint Surg Am.

[20] Engh CA (1983). Hip arthroplasty with a moore prosthesis with porous coating. Clin Orthop Relat Res.

[21] Cappozzo A, Catani F, Della Croce U, Leardini A (1995). Position and orientation of bones during movement: anatomical frame definition and determination. Clin Biomech.

[22] Nishihara S, Sugano N, Nishii T, Ohzono K, Yoshikawa H (2003). Measurements of pelvic flexion angle using three-dimensional computed tomography. Clin Orthop Relat Res.

[23] Murray DW (1993). The definition and measurement of acetabular orientation. J Bone Joint Surg Br.

[24] Montgomery AA, Graham A, Evans PH, Fahey T (2002). Inter-rater agreement in the scoring of abstracts submitted to a primary care research conference. BMC Health Serv Res.

[25] Lewinnek GE, Lewis JL, Tarr R, Compere CL, Zimmerman JR (1978). Dislocation after total hip-replacement arthroplasties. J Bone Joint Surg Am.

[26] Mckibbin B, England S (1970). Anatomical factors in the stability of the hip joint in the newborn. J Bone Joint Surg Br.

[27] Bargar WL, Jamali AA, Nejad AH (2010). Femoral anteversion in THA and its lack of correlation with native acetabular anteversion. Clin Orthop Relat Res.

[28] Reikerås O, Gunderson RB (2011). Components anteversion in primary cementless THA using straight stem and hemispherical cup: a prospective study in 91 hips using CT-scan measurements. Orthop Traumatol Surg Res.

[29] Dorr LD, Wan Z (1998). Causes of and treatment protocol for instability of total hip replacement. Clin Orthop Relat Res.

[30] Berry DJ (2001). Unstable total hip arthroplasty: detailed overview. Instr Course Lect.

